# Epigenetic Contribution of High-Mobility Group A Proteins to Stem Cell Properties

**DOI:** 10.1155/2018/3698078

**Published:** 2018-04-24

**Authors:** Vincenzo Giancotti, Natascha Bergamin, Palmina Cataldi, Claudio Rizzi

**Affiliations:** ^1^Department of Life Science, University of Trieste, Trieste, Italy; ^2^Trieste Proteine Ricerche, Palmanova, Udine, Italy; ^3^Division of Pathology, Azienda Ospedaliero-Universitaria, Udine, Italy

## Abstract

High-mobility group A (HMGA) proteins have been examined to understand their participation as structural epigenetic chromatin factors that confer stem-like properties to embryonic stem cells (ESCs), induced pluripotent stem cells (iPSCs), and cancer stem cells (CSCs). The function of HMGA was evaluated in conjunction with that of other epigenetic factors such as histones and microRNAs (miRs), taking into consideration the posttranscriptional modifications (PTMs) of histones (acetylation and methylation) and DNA methylation. HMGA proteins were coordinated or associated with histone and DNA modification and the expression of the factors related to pluripotency. CSCs showed remarkable differences compared with ESCs and iPSCs.

## 1. Introduction

Three polypeptides HMGA1a, HMGA1b (together HMGA1), and HMGA2 are high-mobility group A nuclear phosphoproteins that are highly expressed in undifferentiated and cancer cells, but that are noticeably absent in adult differentiated cells. Using previous nomenclature, these proteins were identified as HMGI, HMGY, and HMGI-C, respectively. The high levels of expression in embryos, which is followed by a gradual decrease and the need for these genes to remain unaltered, suggest that HMGA proteins play fundamental roles in normal development [1–3].

Why are HMGA proteins considered epigenetic factors?

If epigenetics comprises processes and molecular factors that modify the three-dimensional structure of chromatin without altering the primary sequence of DNA, then HMGA proteins should be considered epigenetic factors because they are architectural elements that modify the global structure of chromatin as well as organizing specific sites of expression in cooperation/competition with histones and in cooperation with other factors involved in epigenetic gene expression processes. If so, HMGA proteins should accompany embryonic stem cells (ESCs) through the various differentiating lineages. ESCs are blastocyst-derived stem cells that show self-renewal and invasion as natural properties, together with pluripotency, that is, the capability to differentiate and give rise to many progressive specific lineages to build a complete organism. ESCs constitute then the logical reference system to interpret two other types of stem cell: induced pluripotent stem cells (iPSCs) and cancer stem cells (CSCs).

iPSCs were artificially produced for the first time by Takahashi and Yamanaka through ectopic expression of Oct4, Sox2, Klf4, and cMyc (together OSKM) in murine somatic cells [4] and by Thompson's group in human cells by replacing Klf4 and cMyc with factors LIN28 and NANOG [5]. LIN28 expression leads directly to the expression of HMGA proteins and the induced cells show properties similar to ESCs, with self-renewal capacity, invasion, and pluripotency of yielding cells useful for regenerative medicine. Since these breakthroughs, many studies have found that induced pluripotency is also feasible by using other methodologies and molecules including HMGA proteins [6–11]. We focused on HMGA proteins in iPSCs because HMGA proteins are as highly expressed in these cells as in ESCs [1–3].

Tumours and cancer cell lines express at least one type of HMGA proteins (HMGA1 or HMGA2) and show a high level of oncogenic transformation [12]. CSCs are a subpopulation of cancer cells that have some characteristics similar to ESCs and iPSCs including self-renewal and invasiveness. Moreover, they exhibit resistance to eradication by therapy; however, currently, their pattern of differentiating into normal cell lineages remains unknown. Although the properties of CSCs are well understood, their origin is controversial; in heterogeneous tumour masses, they represent a small fraction of cells, whose origin is uncertain and which are likely cancer type dependent. In any case, CSCs have been reported to express epithelial-mesenchymal-transition (EMT) factors as well as HMGA proteins, and they should be considered a high oncogenically transformed system [13].

In our previous review [12], we discussed the expression of HMGA proteins and pathways involved in seven types of cancer. We examined, in detail, results obtained by six different research groups that worked on the same breast cancer cell line, MDA-MB-231, which shows a triple-negative phenotype. All the authors agreed on reporting high levels of expression of both HMGA1 and HMGA2 in MDA-MB-231 cells, which have some properties of stem cells (self-renewal and invasion), while the property of metastasis is a specific characteristic of tumour cells. From the analysis of the results from published studies on seven cancers (breast, colorectal, prostate, lung, thyroid, ovarian, and brain), HMGA proteins were found to be derived from many active pathways such as Wnt/*β*-catenin, RAS/RAF, TGF-*β*, PI3K/Akt, and IL-6/Stat3, and, at same time, they induced these pathways, establishing an interconnected and self-stimulating process that drives cells towards high level of oncogenic transformation. These cells, likely CSCs, express high levels of both HMGA1 and HMGA2; this might constitute an essential element of resistant cancer cells characterized by well-defined self-renewal and invasion factors.

Here, we extend the analysis that we carried out on cancer cell lines and tumours to ESCs and iPSCs, because the three types of cells share original factors that constitute an early starting point of ESCs and iPSCs in development and, conversely, a rather stable positioning for CSCs. To this end, we examined only some ESCs varieties among differentiating lineages (because of limitations in the length of the review) and discuss cancer properties and iPSCs.

## 2. Chromatin Epigenetic Network

The main properties of ESCs are self-renewal and pluripotency which allow an increase in the number of stem cells necessary to build the whole organism and differentiation of these cells into all opportune lineages that give rise to all tissues. HMGA proteins are highly expressed in such cells [2].

Self-renewal and pluripotency of ESCs are assured by the presence of a few specific factors such as OCT4, SOX2, KLF4, cMYC, NANOG, and LIN28 whose expression is due to a precise chromatin structure derived from epigenetic modifying events that regulate chromatin organization and, consequently, gene expression in all type of cells. These events include the following:

- DNA methylation (m)/demethylation;
- histone acetylation (Ac)/deacetylation;
- histone methylation (me)/demethylation;
- alteration of the nucleosomal structure;
- regulation of gene expression by microRNAs (miRs) and long noncoding RNAs (lncRNAs).

These events do not occur independently of each other; rather they are connected to confer a precise functional structure to large or small parts of the chromatin.

DNA can be methylated at the cytosine 5-position of CpGs by DNA methyl-transferases (DNMTs). Unmethylated or hypomethylated DNA participates to the formation of an open (or active or unrepressed) euchromatin structure which allows the high levels of gene expression needed for ESCs to differentiate in various lineages. DNA modification (as well as histone modifications) is so determinative that a different degree of methylation can promote differentiation into an alternative lineage. DNA methylation exerts a repressive effect on pluripotency and initiates differentiation. In contrast, demethylated DNA allows iPSCs to acquire pluripotency similar to that of ESCs. Repression of DNA methylation by inhibiting DNMTs preserves the pluripotency of ESCs, while active DNMTs (such as DNMT1) induce the transition from pluripotency to multipotency [14, 15].

Another aspect of the polyhedral regulation of chromatin is posttranscriptional modifications (PTMs) of histones, which mainly consists of acetylation and methylation, particularly in lysines (K) of histone H3. Acetylation of lysines eliminates the positive charges that enable interactions with negatively charged DNA phosphates to compact the chromatin. Therefore, acetylation promotes open or unrepressed chromatin as unmethylated DNA. H3K9Ac, if present in promoters, activates transcription [16]. This modification is associated with the self-renewing capacity and pluripotency of ESCs and iPSCs. Activation of chromatin by H3K9Ac is coupled in the same action by H3K4me2/3: H3K9 hyperacetylation and H3K4 methylation induce the expression of pluripotent genes such as Oct4 and NANOG to maintain self-renewal [17].

Changing acetylated H3K9Ac into methylated H3K9 (H3K9me2/3) results in a closed or repressed heterochromatin structure to which H3K27me3 strongly contributes. Indeed, H3K9me3 is considered a barrier to efficient induction of somatic cells into iPSCs [18]. In ESCs, NANOG and lysine demethylase 1 together repress the genes involved in development, and NANOG shortens the cell cycle length by positively regulating the CDK6 kinase gene in the G1/S transition. The proliferation capability of MSCs before differentiation is guaranteed by the pair of self-renewal factors NANOG/OCT4 [19, 20].

Lysine methylation results from the action of the catalytic subunit EZH2 (enhancer of zest 2) of the polycomb complex 2 (PcG2) [21–27]. Through H3K27me3, EZH2 represses genes involved in both differentiation and cancer. During differentiation, EZH2 allows the transition from pluripotency to multipotency and progressively decreases self-renewal and proliferation up to mature differentiated cells. In cancer, EZH2 does not repress self-renewal that is retained. Although EZH2 is in any case a repressor, it can act differently on the basis of the other factors accompanying it. For example, tumour suppressors such as p16^INK4a^ are repressed in cancer, but activated in differentiated cells [23, 24]. Indeed, Song et al. [28] defined EZH2 as a candidate oncogenic driver in a study on MDA-MB-231 and 4T1 triple-negative breast cancer cells. EZH2 overexpression in triple-negative breast cancer cells was shown to be related to self-renewal, migration, invasion, and tumour suppressor silencing. Consequently, the use of agents such as ZLD 1039, which inhibits EZH2 activity, stops metastasis. Moreover, it was reported that inhibition of the histone deacetylases (HDACs) also shows inhibition similar to that of EZH2 in an anticancer treatment. Here we must mention the striking difference in cancer cells compared to ESCs and iPSCs, in which EZH2 expression and histone deacetylation are associated with differentiation, that is, with a decrease of the proliferation.

As mentioned above, H3K4me3 and H3K27me3 regulate an open or closed (unrepressed or repressed, resp.) chromatin structure. However, these two different modifications of histone H3, which have opposite functions, may be present in the same promoter, referred to as bivalence [29]. There is a functional dualism in which the preponderance of one modification or the other allows the activation or repression of a gene.

The addition or removal of modifications in both DNA and histones needs an alteration of the compact nucleosomal structure that is achieved by the specific remodeling ATP-dependent enzymes SWI/SNF, ISWI, and CHD. These chromatin remodeling agents are also able to change the position of nucleosomes along the DNA sequence modifying then the length of the linker DNA where histone H1 is bound [30–33]. Remodeling factors and PTMs are related. For example, the repression of the remodeling factor Snf5 upregulates H3K27m3 and increases p16^INK4a^ repression in cancer [23, 24, 34].

## 3. Searching for the Location of the HMGA Proteins in the Chromatin Epigenetic Network

### 3.1. Relationships between HMGA Proteins, EZH2, and Proliferation Factors

**Figure 1:**
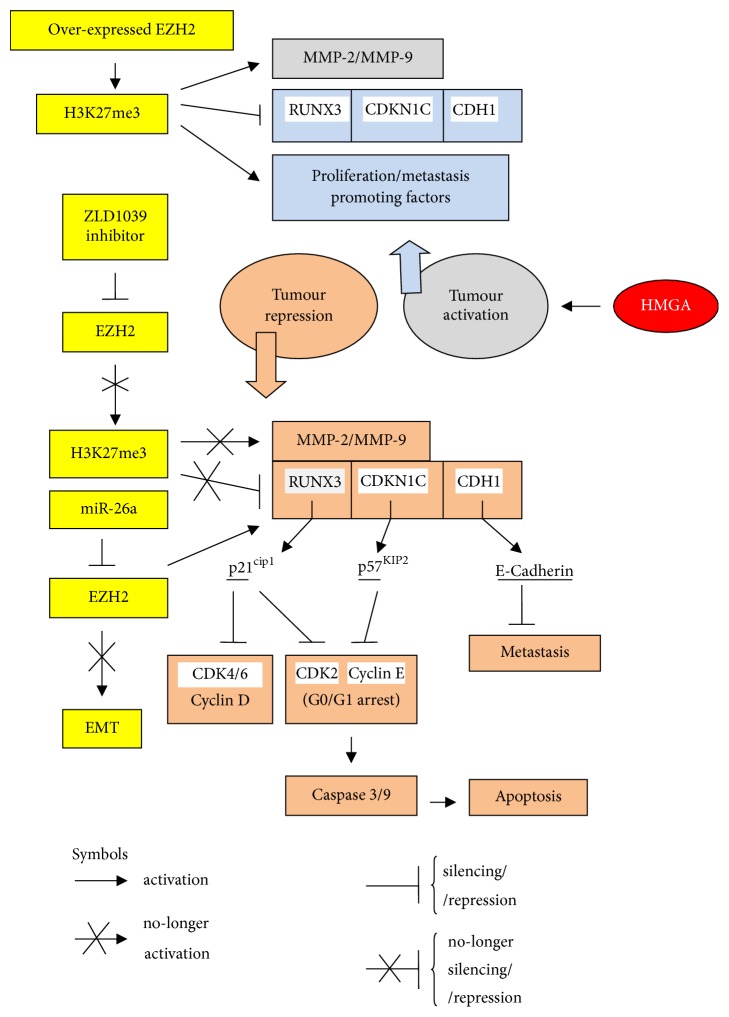
Alternative cascades of events following EZH2 overexpression/repression in cancer cells. CDH: cadherin; CDK: cyclin-dependent kinase; CDKN1C: cyclin-CDK inhibitor p57; EMT: epithelial-mesenchymal-transition; EZH2: enhancer of zeste 2; MMP: metalloprotease; p21^cip1^: p21 CDK-interacting protein 1; p57^KIP2^: p57 kinase inhibitory protein 2; RUNX3: runt-domain transcription factor 3.

**Figure 2:**
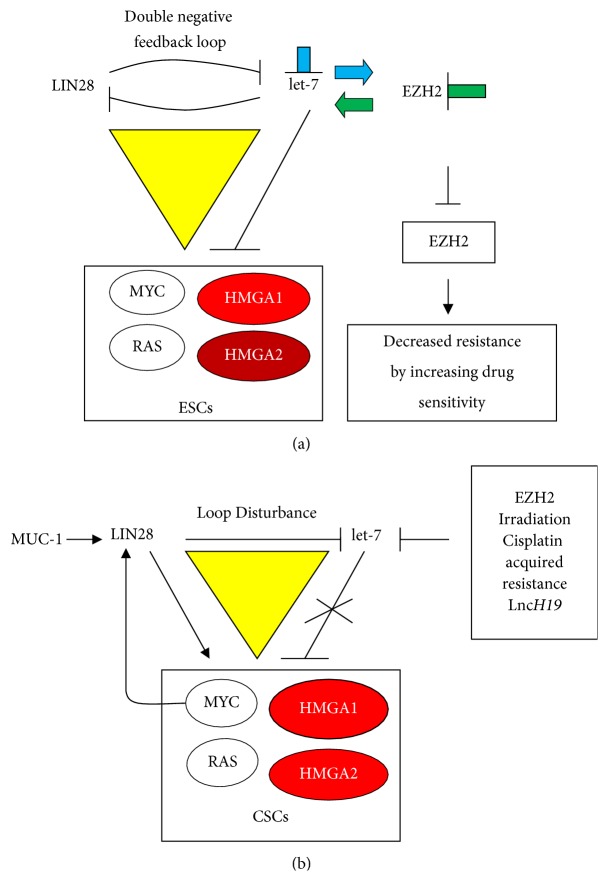
LIN28-miR-let-7 feedback loop and its regulation in ESCs and CSCs. Symbols are the same as shown in Figure 1.

**Figure 3:**
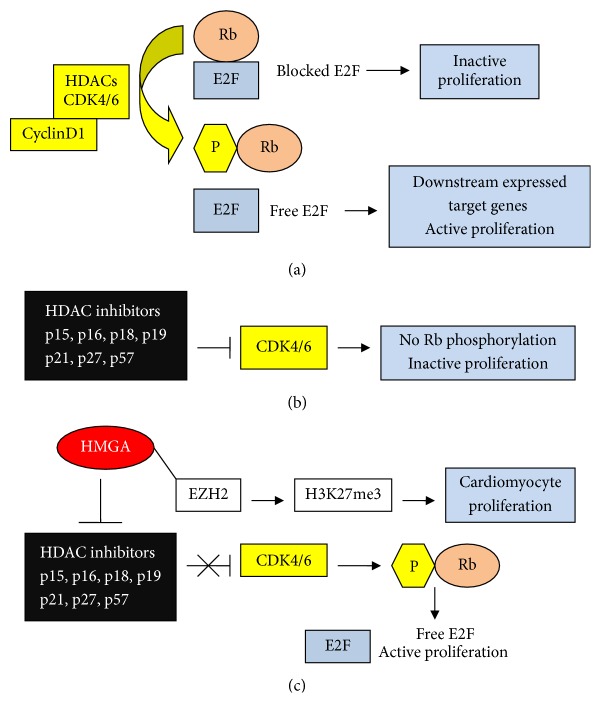
Rb and E2F in CSCs. (a) Phosphorylated Rb dissociates from E2F that induces proliferation factors; (b) blocking of Rb CDK4/6 kinases prevents Rb phosphorylation and inactivates proliferation; (c) HMGA proteins inhibit CDK4/6 Rb kinases and block cycle repressing-factors: proliferation is reactivated. Symbols are the same as shown in Figure 1.

The possible effects of either overexpression or repression of EZH2 in cancers such as breast, bladder, gastric, hepatocellular, lung, thyroid, and tongue are shown in Figure 1. HMGA proteins show actions consistent with those of EZH2 in promoting tumours and proliferation [12, 28, 35–41]. Active EZH2 induces and activates, in conjunction with HMGA, tumour-promoting factors and proliferation, repressing differentiating factors such as runt-domain transcription 3 factor (RUNX3), p57 cyclin-CDK inhibitor 1C (CDKN1C), and cadherin 1 (CDH1) [28, 42–49]. Inhibition of EZH2 by ZLD1039 or miR-26a no longer induces tumour invading factors such as metalloprotease 2 and 9 (MMP2/9) and those related to epithelial-mesenchymal-transition (EMT) and, in contrast, induces differentiating factors. Further support to the connected action of EZH2 and HMGA proteins derives from studies on other cancers in which EZH2 and HMGA (frequently HMGA2) converge towards the oncogenic achievement. In prostate cancer [50–52], EZH2 overexpression correlates with high levels of oncogenic transformation and is due to the loss of miR-let-7, the miRs' family known as the main repressor of HMGA proteins. In breast cancer and non-small cell lung cancer (NSCLC), in which HMGA proteins are overexpressed [12], the protein MUC1-C activates EZH2 promoter through induction of the pRb-E2F pathway [52]. These relationships will be more extensively discussed in Figures 2 and 3. In bladder cancer [38–40], HMGA2 is upregulated and EMT established, while E-cadherin is repressed. Notably, EZH2 also induces EMT and represses E-cadherin promoting metastasis [53, 54]. In conclusion, EZH2, HMGA proteins, and miR-let-7 family are strictly linked in determining the cellular state in which other factors participate, such as the LIN 28 proteins (partners of miR-let-7), as we are going to illustrate in the following paragraphs.

### 3.2. HMGA Proteins and Factors of Pluripotency and Proliferation

The miR-let-7 family and LIN28 proteins have been described as tumour suppressors and tumour inducers, respectively [41, 55–60]. In normal development, the opposing actions of LIN28 and let-7 axis assure proper timing for development, proliferation, and differentiation. In this axis, HMGA2 participates [61–65]. As shown in Figure 2(a), the predominance of let-7 can result from both reduced expression of LIN28 and decreased activity of EZH2, that, in contrast, can be activated by let-7 repression [51, 66]. Increased activity of let-7 allows negative regulation of cancer factors such as RAS, MYC, HMGA1, and HMGA2; in other words, the origin and maintenance of CSCs are impeded [67]. Disturbance of the double-negative feedback loop causes severe effects as shown in Figure 2(b) [66, 68]. Many possible actions can decrease let-7 expression. LIN28 can be overexpressed by oncogenic factors such as MUC-1 or through a feedback loop with MYC [69]. let-7, initially increased by chemotherapeutic treatments, can decrease de novo because of tumour acquired resistance following, for example, irradiation or cisplatin therapy that likely increases EZH2 in human non-small cancer lung cells (NSCLCs) [68]. Consistently, in pancreatic cancer cells, EZH2 depletion decreases resistance to doxorubicin and gemcitabine, allowing p27 expression and apoptosis induction [70, 71]. Moreover, long noncoding RNA can downregulate let-7 and consequently increase LIN28, which is then in a position to induce oncogenesis and establish CSCs [72]. HMGA1 and HMGA2 are deeply implicated in the triangulation of the factors and events shown in Figure 2 because they belong to groups of factors that grant self-renewal capacity to cells. However, it should be noted that Figure 2 presents an incomplete view of the complex relationships that link other factors such as Sox2, [67, 73, 74].

The couple proteins Rb and E2F are a well-known complex involved in proliferation, because E2F induces the expression of target genes that are proliferation factors in cancer. An unphosphorylated (or hypophosphorylated) form of Rb participates in an E2F complex; this status prevents the transcription of E2F-dependent tumour-promoting factors. pRb hyperphosphorylated by cyclin D1/CDK4/6 dissociates from the complex, inducing E2F factors, cell cycle progression, and proliferation (see Figure 3(a)) [75, 76]. Inactivation of the free E2F can result from repression by INK4A family repressors (p15, p16, p18, and p19) and CIP/KIP family repressors (p21, p27, and p57) that prevent the G1/S transition [77–79]. Proteins such as p16 are active in normal tissues but absent in cancer tissues or highly proliferating stem cells. It is worthwhile to mention that enzymes, such as HDACs, which modify histones, are able to modify other proteins. A parallel action (Figure 3(b)) is carried out by HDAC inhibitors, because HDACs are proliferation promoters. For example, HDAC2 is highly expressed in tumours and related to p16; by inhibiting HDAC2, p16 activity is promoted and cells are arrested in G1/S [80]. Similarly, in pituitary tumourigenesis, HMGA2 displaces HDAC1 from the complex Rb/E2F1, leaving the latter in an active acetylated form [81]. We should note that HMGA and HDACs are consistent in inducing proliferation. The schemes in Figures 3(a) and 3(b) may no longer be valid if there are upstream events that activate pathways or modify gene structures (such as mutation and amplification of DNA and histone epigenetic modifications) which could influence the factors under discussion (Figure 3(c)). First, the upstream expression of HMGA1 and HMGA2 could change the effect of linked factors. Indeed, expression of the tumour-promoting HMGA proteins (expressed also in ESCs) represses factors such as p16, whose repressing effect is reversed and, consequently, that of Rb [82–85]. Indeed, the active* Ink4a/Arf* locus, which expresses p16 and p19, is blocked by HMGA2, which in turn is repressed by miR-let-7b; elevated expression of miR-let-7b reduces the self-renewal capacity of neuronal stem cells (NSCs) [86]. Finally, a parallel action, shown in Figure 3(c), is exerted in mice by EZH2 according to the paper by He et al. [87]. EZH2 represses p16^Ink4a^ through H3K27me3 resulting in CDK4/6 upregulation and cardiomyocyte proliferation.

### 3.3. HMGA Proteins in Adipogenesis and Osteogenesis

**Figure 4:**
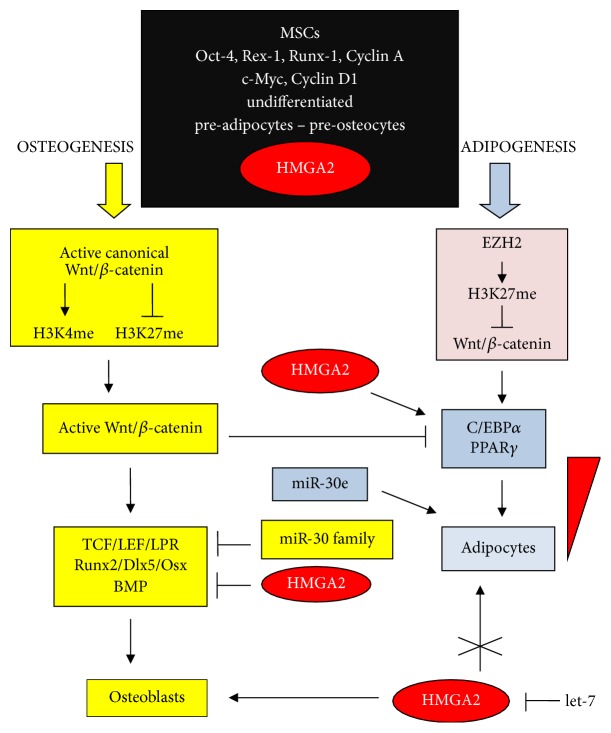
Alternative adipocyte and osteocyte differentiating pathways of MSCs based on EZH2 and Wnt/*β*-catenin actions. BMP: bone morphogenetic protein; C/EBP: CCAAT/enhancer binding protein; Dlx: distal-less homeobox; LPR: low-density lipoprotein receptor related protein; Osx: Osterix; PPAR*γ*: peroxisome proliferator-activated receptor *γ*; Runx: runt-related transcription factor. Symbols are the same as shown in Figure 1.

In MSC differentiation, the canonical Wnt/*β*-catenin pathway works with DNA and histone modifications and factors specific for MSC differentiation [88]. Interestingly, alternative differentiation of MSCs into adipocytes or osteocytes occurs, depending on different combinations of histone H3 modifications with active or repressed Wnt/*β*-catenin that involves gene activation/repression leading to adipogenesis or osteogenesis [89]. The predifferentiation stage of MSCs is characterized by an active canonical Wnt/*β*-catenin pathway with H3K4me and without H3K27me, which gives rise to open chromatin expressing c-Myc and cyclin D1. The process of differentiation can follow two paths, depending on the Wnt/*β*-catenin status [90–93]. If the Wnt/*β*-catenin pathway remains active, *β*-catenin translocated to the nucleus activates the expression of TCF/LEF dependent genes specific for osteogenic differentiation such as Runx2, Dlx5, and Osterix, and at the same time the adipogenic genes C/EBP*α* and PPAR*γ* are repressed [94–100]. In contrast, if high levels of EZH2 are present, Wnt/*β*-catenin is repressed by H3K27me, while the expression of C/EBP*α* and PPAR*γ* is activated [85, 93].  Figure 4 summarizes the progress of the two differentiating lineages. HMGA2 protein induces adipogenesis rather than osteogenesis [101]. However, it is interesting to note that the data in Figure 4 reflects normal development, whereas, in cancer, the Wnt/*β*-catenin pathway and HMGA expression are always consistent; that is, they serve as tumour promoters. In other words, the processes underlying both differentiation and cancer show the presence of repressive epigenetic factors that are apparently contradictory, considering the enormous differences between the two phenotypes. It is evident that repression in the two systems does not follow the same repressive gene pattern. In adipogenesis, HMGA2 is involved in two functions [101, 102]. On the one hand, it guarantees that undifferentiated preadipocytes from MSCs have an open chromatin structure that is needed to initiate differentiation. To this end, HMGA2 activates factors in the C/EBP family and PPAR-*γ* and, at the same time, EZH2 induces H3K27me3, repressing Wnt/*β*-catenin, which is needed because this pathway is an osteogenic promoter rather than an adipogenic one. On the other hand, HMGA2 in conjunction with the STAT3 pathway allows proliferation to produce fat masses [102].

Both adipogenesis and osteogenesis are strongly miR-dependent; however, in Figure 4, we indicate only the miR-30 family among a myriad of miRs discussed elsewhere [103]. Members of the let-7 family of miRs are strong repressors of HMGA proteins, as in cancer [12]. The repression of HMGA2 by let-7 (and other miRs) strongly promotes osteogenesis and inhibits adipogenesis [104, 105] and is linked to both Wnt/*β*-catenin and EZH2 as shown in Figure 4. HMGA2, present in both preadipocytes and preosteocytes, guarantees an open chromatin structure that initiates the two lineages through the factors introduced above. HMGA2 disappears soon after this in osteogenesis, whereas, in adipogenesis, it gradually decreases over time. It is absent in mature differentiated cells, but still present in stem cells that constitute the reserve for replacing dead cells. However, HMGA2 repression by let-7 allows osteogenesis to proceed, while adipogenesis is repressed because C/EBP and PPAR-*γ* are not activated.

We have focused our discussion on the differentiation of MSCs from the mesoderm based on the factors introduced above.  Table 1 summarizes in a concise form the relationships between the Wnt pathway, miR-let-7, HMGA2, and EZH2 which are involved in MSCs differentiation, beginning from mesoderm and progressing to four mature differentiated cells: adipocytes, osteocytes, myocytes, and cardiocytes. The marks (+) and (−), indicating a positive or a negative contribution, respectively, to the differentiating process, are rather simplistic and incomplete in showing a complex program that is characterized by progressive changes, with factors from each stage still expressed in subsequent stages. In other words, differentiation and development are frequently used in a generic manner, although they refer to different and overlapping processes: from pluripotency to multipotency and monopotency (of stem cells as the reserves to regenerate tissues); from proliferation and invasion to the maturation of nonproliferating cells; and consequently from factors and pathways of pluripotency and invasion to molecules that are characteristic of differentiated cells. As shown in Table 1, it can be difficult to determine the precise point of action of these factors.

### 3.4. HMGA Proteins in Myogenesis

Adipogenesis and osteogenesis, initiated by MSCs, are discussed above. Myogenesis and osteogenesis deserve additional comments.

**Figure 5:**
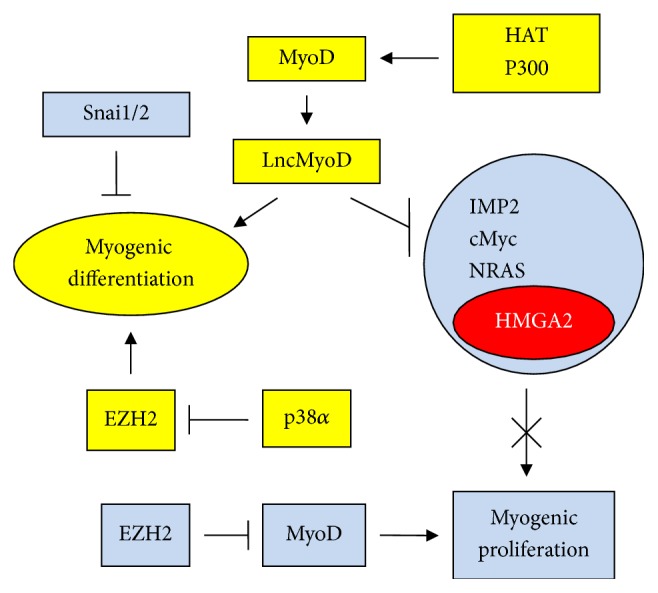
Relationship between MyoD, pluripotency factors, and EZH2 in myogenic differentiation. Symbols are the same as shown in Figure 1.

Pluripotency and proliferation of ESCs are assured by factors such as IMP2, cMyc, NRAS, and HMGA2. MyoD is a factor of myogenic differentiation that represses proliferation through long noncoding MyoD RNA (LncMyoD) [106] once a proper number of cells to be terminally differentiated are produced. It is conceivable that HMGA2 is repressed because its expression is strictly associated with the above factors (Figure 5, yellow). The repression of pluripotency factors indicates the end of myogenic proliferation. In contrast, the EMT factors Snai1/2 repress myogenic differentiation because they are associated with the invasion and lack of differentiation of cells [107, 108] (Figure 5, blue). MyoD repression by EZH2 stimulates proliferation [109], while EZH2 degradation in response to the phosphorylation of p38*α* kinase arrests proliferation, allowing differentiation to prevail [110] (Figure 5). Inhibition of EZH2 decreases H3K27me3 modifications, and the transition from MSCs to differentiated cartilage is increased [111]. Moreover, in differentiation, MyoD factor is acetylated; HAT p300 acetylates MyoD during myogenic differentiation and increases its transcriptional activity [112, 113]. It is interesting to note that EZH2 repression (and consequently H3K27me3 repression) induces osteogenic and myogenic differentiation and suppresses tumour formation. Wnt3a is one of the ligands that can induce the canonical *β*-catenin pathway [114]. The expression of HMGA proteins and proliferation are induced through the association of *β*-catenin, TCF, and LEF [115–117]. In myogenesis, this occurs early for later differentiation of cells. Wnt3a action (early stage) overlaps the initiation of MyoD expression, when Wnt3a activity should be ending [118–120]. In Table 1, these two states are shown.

**Figure 6:**
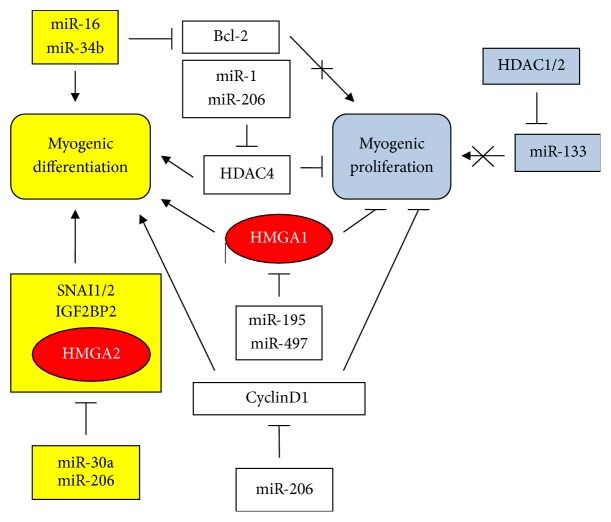
miRs, HDACs, and HMGA proteins in myogenic differentiation. Left side (yellow): induction of differentiation; right side (blue): suppression of proliferation. Symbols are the same as shown in Figure 1.

A large number of miRs involved in myogenic differentiation have been reported. Horak et al. [121] introduced a list of miRs involved in skeletal muscle development. Among these miRs, we show the action of miR-1, miR-133, and miR-206 in Figure 6. These miRs, also reported by Chen et al. [122], are also involved in myogenesis together with miR-34b [123], miR-16 [124], and miR-195/497 [125]. The middle of Figure 6 shows the contribution of various miRs to the promotion of myogenic differentiation (left side, yellow) which results in the inhibition of myogenic proliferation (right side, blue). The activating/repressing events are rather complex. miR-1 and miR-206 downregulate histone deacetylase 4 (HDAC4). Inhibition of deacetylases reduces proliferation of cancer cells. Consequently, as shown in Figure 6, HDAC4 inhibition promotes myogenic differentiation. According to the study by Chen et al. [122] proliferation and differentiation are mutually exclusive in skeletal muscle formation in which miR-1 and miR-206 are inducers of differentiation while miR-133 is an inducer of proliferation, assuming it is not blocked by HDAC1/2 (Figure 6). In this context, HMGA2 protein is involved in tissue regeneration because its expression expands muscle proliferating myoblast progenitors [126]. Moreover, HMGA2 targets IGF2BP2 (also named IMP2), which in turn induces many genes that promote cell growth, including cMyc and SP1. For example, IMP2 and its homolog IMP1 are involved in neuronal precursor cell proliferation, along with HMGA2; in adult neuronal stem cells, let-7 downregulates IMP proteins and HMGA2 [83, 127–129]. Finally, the inhibition of HMGA1 (a self-renewal factor) by miRs 195/497 and that of cyclin D1 (a cell cycle promoter) by miR-206 induce differentiation (left) and downregulation of proliferation (right) (Figure 6).

Many transcription factors allow stem cells to be either normal or cancerous. Snail1 and Slug (also named Snail2), are some of these transcription molecules. Indeed, Snail1 and Slug, by repressing the membrane protein claudin-1, activate EMT in both normal canine kidney cells (MDCK) and MDA-MB-231 breast cancer cells [130]. In Figure 6, we show that cells engaged in differentiation should lose their invasion capability, which is a property of self-renewing cells. miR-30a and miR-206 downregulate, in myogenic differentiation, Snai1/2 which are associated with stemness as above discussed. Finally, in the upper part of Figure 6, the action of Bcl-2 is illustrated. This is an antiapoptotic agent and a general inducer of proliferation. There is a four-side relationship that links Bcl-2, HMGA2, p53, and miR-34a in which p53 is a positive inducer of miR-34a which, in turn, inhibits Bcl-2. In contrast, HMGA2 is an inducer of Bcl-2 and, consequently, proliferation [131–135]. In myogenesis, the downregulation of Bcl-2 by miR-16 and miR-34b [123, 124] results in the inhibition of myogenic proliferation (Figure 6).

**Figure 7:**
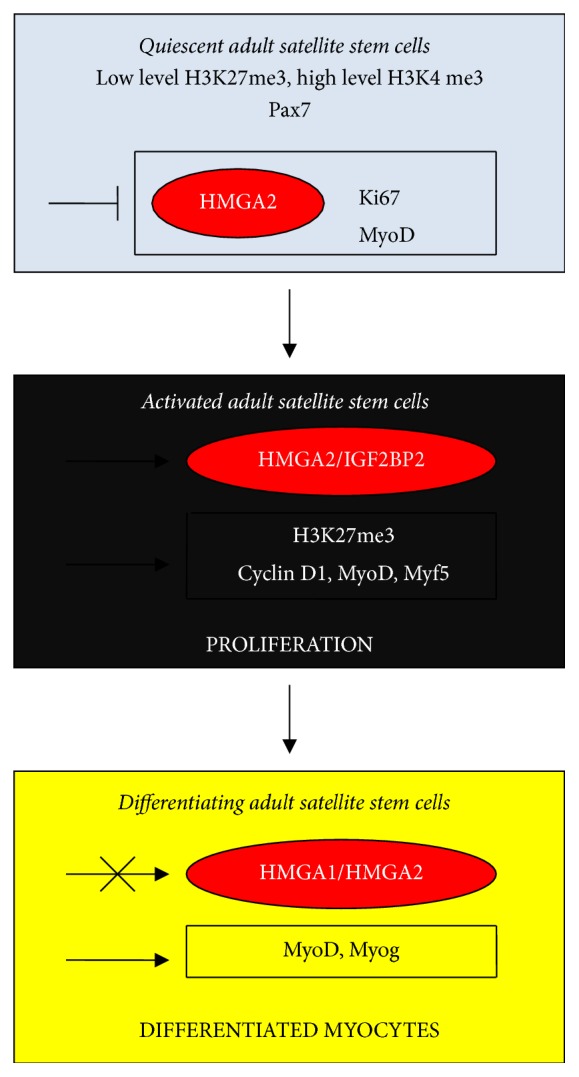
From quiescent adult satellite stem cells to differentiated myocytes. Symbols are the same as shown in Figure 1.

To better understand the location of HMGA proteins in myogenesis, we examined their action in satellite stem cells, which are postnatal stem cell stock for muscle regeneration. If this function is not required, satellite stem cells remain in an nonproliferating quiescent state (Figure 7) that is characterized by Pax7 [136], a known satellite stem cell marker, the repression of the growing factor HMGA2, and the absence of both the proliferating index Ki67 and the differentiation-related factor MyoD [137]. Chromatin is in an open and permissive state because of histone H3 modifications and is ready to receive environmental information to activate development [136–138]. Once factors in the microenvironment are produced in response to a request for regeneration, satellite stem cells are activated by the proliferation-inducing HMGA2/IGF2BP2, the cell cycle inducer cyclin D1, and an increase in H3K27me3. Many specific myogenic factors such as MyoD and Myf5 are expressed and growth starts [125, 139, 140]. Once an appropriate number of cells have been produced and the action of HMGA proteins is no longer necessary, their expression is repressed as specifically reported for HMGA1 [141, 142], and differentiation proceeds to completion. In conclusion, the data shown in Figures 5–7 suggest the involvement of HMGA proteins, in conjunction with many other factors, to produce proliferating cells that can be differentiated from myogenic stem cells. Because members of the let-7 family of miRs are important repressors of HMGA protein (and other self-renewal factors) expression, Table 1 shows a positive (+) contribution for let-7 that indicates differentiation, while the contribution is negative (−) for proliferation.

### 3.5. HMGA Proteins in Cardiomyogenesis

**Figure 8:**
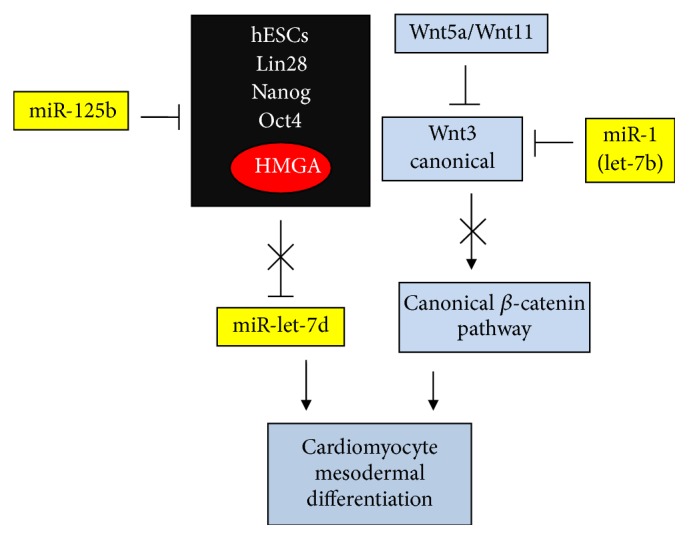
From hESCs to cardiomyocyte differentiation through miRs and Wnt activities. Symbols are the same as shown in Figure 1.

miR-let-7 overexpression is required for human embryonic stem cell-derived cardiomyocytes (hESCs, CM) [143]. To this end, as shown in Figure 8, self-renewal/proliferation factors such as Lin28, NANOG, Oct4, and HMGA2 that characterize hESCs should be repressed, for example, by miR-125b. miR-125b overexpression, by downregulating self-renewal factors, allows unrepressed let-7 to induce differentiation to cardiac muscle cells [144].

As in myogenesis, in cardiogenesis, Wnt3a activates proliferation if the *β*-catenin pentadegradating complex is inactive because of the modification of one or more of its component such as CK1. *β*-catenin then accumulates in the nucleus, where, in association with TCF/LEF, it induces specific gene expression for proliferation. If the pentacomplex is active in degrading *β*-catenin, then proliferation is hampered and cardiomyocytes are activated for differentiation [145]. miR-1 is the main regulator of vertebrate cardiomyogenesis [146]; its overexpression promotes differentiation of cardiomyocytes from multipotent MSCs by downregulation of Wnt3a, which is a canonical inducer of proliferation (Figure 8). Lu et al. [146] also report that the expression of let-7b in cardiomyocytes (CM) is similar to that of miR-1. The conclusion is that, considering only the differentiation stage, there is a positive (+) contribution by let-7 and a negative (−) one by Wnt, as in myogenesis (Table 1). Notably, some ligands, such as Wnt-5a and Wnt-11, act as repressors of canonical *β*-catenin signaling promoting the differentiation of cardiac progenitors [147–149].

**Figure 9:**
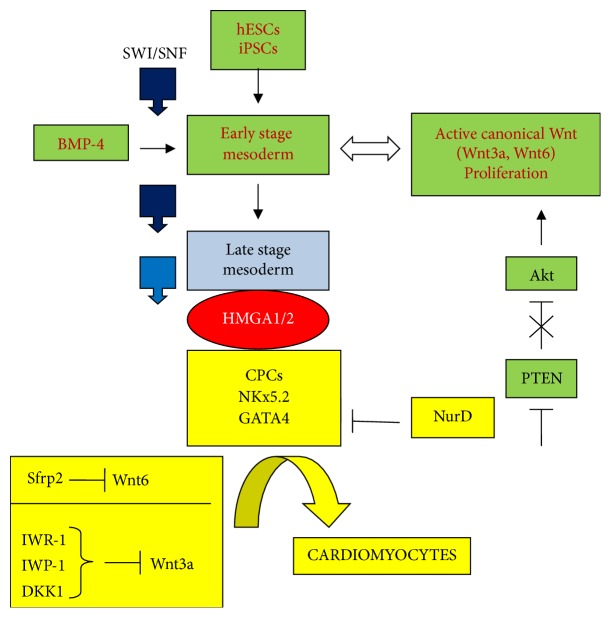
Wnt pathway repression in cardiomyocyte differentiation: from ESCs to cardiomyocytes through cardiac progenitor cells (CPCs). Symbols are the same as shown in Figure 1.

The transition from hESCs or iPSCs could occur, for example, as a result of exposure to bone morphologic protein-4 (BMP-4) [150], which induces an early mesodermal differentiation stage by repressing SOX2 and promoting SLUG, MSX2, and EMT. At this stage, cells still show proliferation properties; however, these are specifically directed towards cardiomyocyte production (Figure 9). Pluripotent stem cells factors such as SOX2 are repressed and the canonical Wnt pathway is responsible for proliferation. Indeed, an active Akt signal (because its repressor PTEN has been deleted) induces the *β*-catenin pathway which promotes proliferation of cardiac progenitor cells [151]. To activate late cardiomyocyte differentiation of already proliferating cells, it is necessary to block Wnt signaling. To this end, there are many choices: protein factors such as secreted frizzled related protein 2 (Sfrp2), dickkopf protein 1 (DKK1), or synthetic chemical compounds (such as IWR-1 and IWP-1) can inhibit Wnt [152–156].

Figure 9 indicates Gata4 and NKx2.5, as two factors that characterize cardiogenic differentiation. GATA4 in an acetylated form (as MyoD in myogenesis) that promotes cardiogenic differentiation. Nucleosomal remodeling and deacetylase (NuRD) is able to deacetylase GATA4 that, in this form, does not induce cardiomyocyte differentiation. Indeed, deacetylases support proliferation rather than differentiation [157]. NKx2.5 is positively regulated by HMGA2 through Smad1/4 of the TGF-*β* pathway and it is a crucial factor for cardiogenesis [158]. Phosphorylated NKx2.5 by p38*γ* kinase is translocated into the nucleus and, together with GATA4, forms a protein complex that is critical for cardiomyocyte differentiation because it maintains the cardiac progenitor cells (CPCs) state. In this context, NKx2.5 enhancer contains modified H3 forms: H3K9Ac, H3K27Ac, and H3K4me3 [159–161]. Notably, NKx2.5 is not expressed in undifferentiated hiPSCs that, in contrast, express Oct4, one of the canonical factors of pluripotency. The induction of NKx2.5 and GATA4 expression requires chromatin modifications of the enhancers by SWI/SNF, whose ATPase Brg1 is a main component of the modifying complex, by HMGA1, and by modified forms of histone H3 (see above) [160, 162, 163]. The SWI/SNF machinery is constantly modifying the chromatin from ESCs until the cells are differentiated, and in the proliferative state they are accompanied by HMGA proteins. However, during development, SWI/SNF activity is progressively modulated by different SWI/SNF subunits, DNA modification, posttransnational protein modifications, and miR action. For example, the change from H3K27me3 to H3K27Ac regulates the change from an inactive gene to an active one in CPCs.

### 3.6. Direct Involvement of HMGA Proteins in Stem Cell Induction and Maintenance

iPSCs develop because of the ectopic expression of pluripotent ESC factors; it is therefore consistent that HMGA proteins are expressed in iPSCs as well as in ESCs. Accordingly, both HMGA1 and HMGA2 have been shown to be highly expressed in iPSCs and to contribute to reprogramming efficiency [10, 164, 165].

OCT4, SOX2, and NANOG maintain the undifferentiated state of ESCs and, if expressed in these cells, of iPSCs and CSCs [166]. The three factors also guarantee pluripotency of ESCs and possibly of iPSCs, but pluripotency of CSCs is questionable if it means these cells are capable of differentiating into normal cells. The three factors are DNA-binding proteins similar to HMGA and histones; however, these factors show secondary and tertiary structures that are different from those of HMGA proteins which are considered unstructured/disordered polypeptides [167, 168]. OCT4 with a helix-turn-helix (H-T-H) containing domain, SOX2 with an HMGB-box containing domain, and NANOG with a homeodomain (HD) containing domain cooperatively bind to the DNA, altering the bending, kinking, looping, and unwinding that allow the action of other factors on the chromatin. The three factors interact with the DNA at the major groove (OCT4), at the minor groove (SOX2), and at both grooves and the DNA backbone (NANOG) to recognize specific DNA sequences and AT-rich regions [169–171]. Interestingly, HMGA proteins recognize as well AT-rich DNA sequences but not in a specific way. Because HMGA proteins are the most abundant proteins bound to DNA after histones and they occupy a large portion of the chromatin, it is conceivable that HMGA proteins predispose the chromatin to receive OCT4, SOX2, and NANOG by sliding along the DNA to find their specific binding sequences. This is much more than a hypothesis because Shah et al. in hESCs showed that HMGA1 binds directly to cMYC, Sox2, and Lin28 promoters and induces the expression of these proteins [10].

Previously, we hypothesized that the expression of HMGA is related to the high level of cell transformation and resistance, in other words to CSCs [12]. Indeed, we found that the expression of HMGA2, although absent in some samples of colorectal cancer (CRC), correlates with cell budding and vascular invasion [172]. Moreover, Kaur et al. [8] showed that HMGA2 is expressed in primary glioblastoma tumours and that its expression strongly correlates with CD133+ expression, a marker of stemness. The authors concluded that HMGA2 should be considered as a stem-like factor of glioblastoma cells, guaranteeing clonogenicity, invasion, and malignant properties. Further support is found in a recent paper by Sun et al. [11] in which it was reported that “HMGA2 increased the expression of the stem cell markers CD44, ALDH1, Sox2, and Oct4 and the EMT-related factors Snail and *β*-catenin” in gastric cancer cells.

As mentioned briefly above, many studies have shown the possibility of obtaining iPSCs using molecules other than Yamanaka OSKM [4] or Thomson OS-LIN28-NANOG factors [5]. Among these molecules are HMGA proteins, whose expression is strongly associated with that of LIN28. Therefore, it should be possible to obtain iPSCs through HMGA. Indeed, Shah et al. [10] first demonstrated this possibility in an exhaustive study on HMGA1. The report showed the following:

1. The expression of HMGA1 induces reprogramming in adult somatic cells to an undifferentiated phenotype with pluripotency characteristic of iPSCs.
2. Differentiating hESCs show decreased expression of HMGA1, along with a decrease in pluripotency factors OCT4, SOX2, and NANOG, which suggests that HMGA1 maintains the undifferentiated state of hESCs.
3. Hyperexpressing HMGA1 in hESCs (that already express HMGA) not only blocks differentiation further, but also increases the levels of pluripotent gene OCT4, SOX2, NANOG, and cMYC expression. The same type of experiment in MSCs showed higher expression of LIN28 (among other factors), demonstrating a feedback loop between HMGA1 and LIN28, which suggests that the loop between cMYC and LIN28 shown in Figure 2 may be valid for all master reprogramming factors.
4. If HMGA1 is overexpressed in somatic cells already transinduced with OSKM factors, the reprogramming rate is increased, stem cells survive, and proliferation is observed, whereas, following HMGA1 knockdown, OSKM factors are repressed.

**Figure 10:**
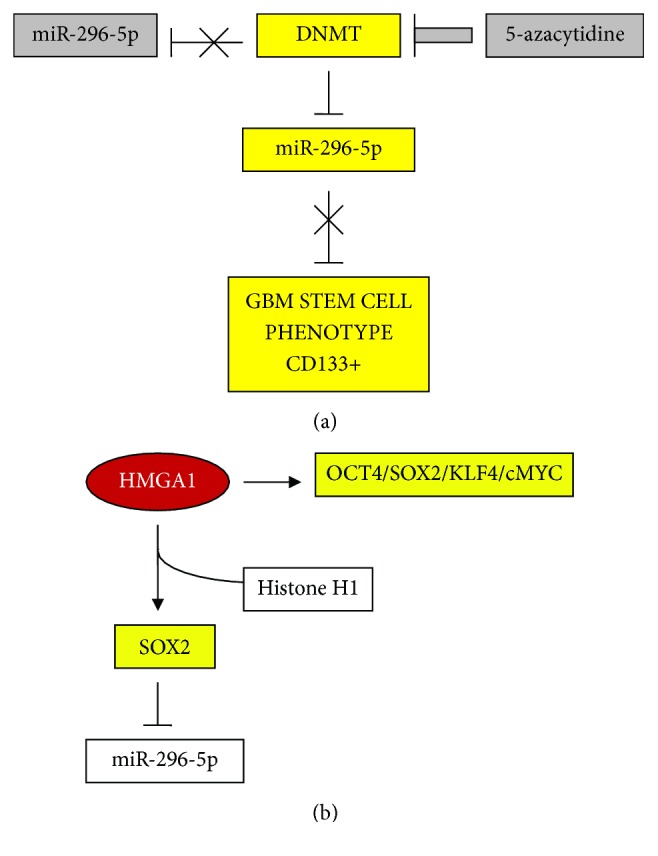
HMGA1 participation in stem cell establishment. (a) DNMTs repress miR-296-5p which can no longer repress the GBM stem cell phenotype. The inhibition of DNMTs by 5-azacytidine no longer blocks miR-296-5p and stem cells are not induced. (b) HMGA1 stimulates the expression of four master pluripotent factors and, with particular efficiency, SOX2 which inhibits miR-296-5p and allows stem cell establishment. Symbols are the same as shown in Figure 1.

A subsequent study on HMGA1 [173] in glioblastoma (GBM) stem cells (SCs) confirmed the above relationship between HMGA1 and pluripotency factors and added evidence of the epigenetic contribution of HMGA1 in SCs. This study focused on the axis between HMGA1/pluripotency factors and miR-296-5p; the results are summarized in Figure 10. This miR is a repressor of the stem cell phenotype in GBM; however, its action is abolished by the repression of its promoter by DNMT methylation. Repression of DNMT by 5-azacytidine reactivates miR-296-5p. On the other hand, HMGA1, which displaces histone H1 from promoters, induces pluripotency factors and particularly SOX2, which represses miR-296-5p. Interestingly, HMGA2 is not involved in miR-296-5p regulation, while the members of miR-let7 family are associated with both proteins.

Two papers from the same research group [174, 175] indicated that it is possible to directly and efficiently reprogram various somatic cells into human induced neural stem cells (hiNSCs) by coexpressing SOX2 and HMGA2. Reprogramming is hampered by miR-let-7b, a member of the well-known HMGA repressor family. Interestingly, if the reprogramming is carried out in the umbilical cord blood derived MSCs that already express HMGA2, reprogramming occurs more easily than in somatic cells.

## 4. Conclusion

The present review is not focused on ESCs, iPSCs, and CSCs for which there are exhaustive reports, some of which we referred to here. Rather, we aimed to elucidate stem cell systems to examine the contribution of HMGA proteins. HMGA proteins are highly expressed in all three systems and related to the structure of chromatin (as already known), as well as global organization and specific gene expression/repression that regulate the development, self-renewal, proliferation, invasion, and EMT of normal and cancer cells. Because of the above discussed properties, it is logical that they have been considered for targeting in cancer therapy. The link between HMGA proteins, CSCs, and drug resistance was further established and HMGA identified as target for sensitizing cancer cells to drug treatments [176, 177]. Cordycepin reduced the expression of the EMT factors in 72 melanoma patient samples comprising HMGA2, Twist1, and ZEB1, and the inhibition of HMGA2 sensitized gastric cancer cells to chemotherapy treatment [178, 179]. A codelivery therapy that inhibits HMGA2 by siRNA and acts on DNA by doxorubicin showed efficacy in CRC and the same dual treatment inhibits cell growth, vimentin (stemness marker), and MMP9 (invasion marker) in breast cancer cells [180, 181]. More studies should be carried out using combination of more drugs focusing on the finding that inhibition of HMGA proteins reduces the resistance of cancer cells to the treatment.

We took into consideration only two histone PTMs, that is, acetylation and methylation, omitting other modifications such as phosphorylation. Similarly, we did not discuss the PTMs by HMGA (although existing), because papers we reviewed did not discuss their modifications.

**Figure 11:**
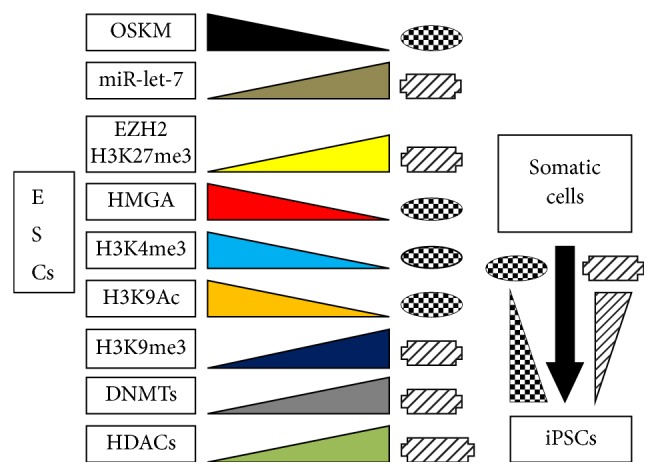
Changes in the action of factors from ESCs to somatic cell (coloured triangles) and reversed action (black arrow) from somatic cells to iPSCs.

Though HMGA proteins are present in all three stem systems, their biological contributions to the maintenance and development of stemness of ESCs, iPSC, and CSCs are quite different, as we discussed at many points above. Established ESCs and iPSCs are considered similar and are characterized by the expression of the same factors, including HMGA, even though they are formed differently. Indeed, whereas ESCs are the natural product of the blastocyst system and do not undergo a preceding differentiation state, iPSCs are derived from somatic cells or from cells having a lower degree of stemness and with the so-called* memory* of the starting state that could reemerge in certain conditions. HMGA proteins are reexpressed in iPSCs but are absent in differentiated cells. However, as shown in Figure 11, the same cooperating set of epigenetic factors are utilized by ESCs to differentiate and, in opposite way, by somatic cells to form iPSCs. Considering these factors, it should be evident that each of them does not work alone; rather, they operate in concert with the other shown factors in a reciprocally modulated fashion, to which other factors not reported in Figure 11 also contribute. For example, the enzyme EZH2 is a component of a complex containing many other molecules that ensure its enzymatic activity. Consequently, an open chromatin structure does not imply the absence of repressed genes and, in contrast, closed chromatin does not imply their presence. Moreover, context-dependent differences, that can modulate or even reverse a preceding result are well established. In other words, HMGA proteins expressed in different stem cells do not necessarily have the same function. HMGA proteins are expressed at every stage in ESC development, throughout which the capacity to proliferate is still needed, including in reactivated quiescent stem cells in reservoir niches. The expression of HMGA proteins is silent once the cells differentiate and mature. Presumably, iPSCs follow a similar pathway of differentiation, but no data are available. Histone acetylation is one among the main modes of epigenetic modification and is associated with HMGA. Acetylated histones accompany HMGA expression in both ESCs and iPSCs and likely in chromatin regions with active gene expression. The deacetylation of histones by HDACs restores the positive charge of lysines, strengthens interactions with the DNA, and increases the compactness of chromatin where HDACs act. In these regions, the expression of reprogramming factors is more difficult (as in somatic cells), and, in order to obtain iPSCs, histones must be acetylated as in ESCs and have consistently high levels of HMGA proteins (Figure 11). Histone deacetylase inhibitors (HDACi) leave the chromatin in an open state as needed for the expression of pluripotent/self-renewal factors, then HMGA and HDACi are able to function in conjunction; consistently, H3K9Ac is present in both ESCs and iPSCc. However, in cancers HDACi are used as antiproliferative agents; that is, an anticancer action requires acetylated histones that are associated with an open state chromatin and the expression of HMGA, which should decrease with anticancer treatments. The difference between ESCs/iPSCs and CSCs is evident and likely involves modes of action with other epigenetic factors and modifications such as methylation. Differentiation of ESCs results from the repressive action of H3K27me3, modified by the enzyme EZH2. However, methylation cannot be considered a repressive modification itself, because H3K4me3 contributes to an open state of the chromatin (Figure 11). On the one hand, there are HMGA, H3K9Ac, and H3K4me3, and, on the other hand, there are H3K27me3, no HMGA, and H3K9me3. An aberrant situation was found in cancer, where H3K27me3 and HMGA are both present; in this case, EZH2/H3K27me3 inhibitors have been suggested as anticancer therapeutic agents. The difference in expression of EZH2/H3K27me3 in ESCs and iPSCs (low level) in comparison with CSCs (high level) extends even to cancer systems. Two examples are illustrative. Luo et al. [182] reported that EZH2/H3K27me3 promotes invasion, metastasis, and EMT of laryngeal squamous carcinoma cells and, at the same time, represses E-cadherin. This is consistent with the above information. However, Cardenas et al. [183] reported that EZH2 inhibition (not expression) promotes EMT in ovarian cancer cells, whereas its expression represses ZEB2, which is a main EMT promoting factor [12]. Both tumours express HMGA proteins [184–186]. In addition, Yi et al. [187] reported that EZH2 promotes ovarian cancer migration and invasion by inhibiting a repressor of MMP2/9, which are tumour promoters (as shown in Figure 1).

DNA methylation by DNMTs has a repressive action on chromatin, similar to that of miR-let-7 family members. In contrast, demethylated DNA is present in ESCs and promotes iPSCs (open chromatin). Demethylated DNA has an important role in the reactivation of genes that induce pluripotency. Indeed, the cooccurrence of H3K9me3 and methylated DNA results in incomplete reprogramming. In contrast, the methylation of DNA by an active DNMT1 is crucial for the transition from pluripotency to multipotency. Demethylation can result from the presence of the enzyme TET1, which catalyses the transformation of 5mC into 5hmC (5-hydroxymethylcytosine) and subsequent formation of unmethylated cytosine [188]. The loss of TET1 leaves methylated DNA, that is, repressed chromatin, which induces cell migration and E-cadherin repression via EZH2/H3K27me3 in colon cancer cells [189]. Once again, we note an aberrant association between epigenetic factors in comparison with that normally found in ESCs and iPSCs.

In conclusion, we examined HMGA proteins, which are well-known cancer promoters, and compared their functions in ESCs and iPSCs as epigenetic chromatin-modifying factors associated with self-renewal, proliferation, invasion, and EMT stemness properties. HMGA proteins are involved at every stage of epigenetic stem cell regulation, up to the last moment when proliferation is required. Their levels of expression can remain the same or differ with the coexpression/modification of other factors, which are linked through a multicomponent molecular machinery that manages chromatin accessibility, first in the formation/maintenance of ESCs, iPSCs, and CSCs and subsequently in the use of these systems based on the specific biological context. We examined how DNA accessibility and gene expression are dependent on a multifactorial machinery whose composition could explain the contradictory results deriving from considering the action of only one factor. This may be expressed in the saying:* one swallow does not make a summer*.

## Figures and Tables

**Table 1:** Wnt/*β*-catenin, miR-let-7, HMGA2, and EZH2 action in mesenchymal stem cells (MSCs) differentiation.

Differentiation lineage	Canonical Wnt	miR let-7	HMGA2	EZH2
Adipogenesis	−	−	+	+
Osteogenesis	+	+	−	−
Myogenesis				
Early	+	+	−	−
Late	−			
Cardiogenesis	−	+	−	−

(**+**): positive contribution; (−): negative contribution.

## Abbreviations

Akt: Serine/threonine kinase 1
BMP: Bone morphogenetic protein
CDH: Cadherin
CDK: Cyclin-dependent kinase
CDKN1C: p57 Cyclin-CDK inhibitor
C/EBP: CCAAT/enhancer binding protein
CM: Cardiomyocytes
CSCs: Cancer stem cells
Dkk-1: Wnt inhibitor dickkopf homolog 1
Dlx: Distal-less homeobox
DNMT: DNA methyl transferase
DNMTi: DNA methyl transferase inhibitor
E2F: E2 transcription factor
EMT: Epithelial-mesenchymal-transition
ESCs: Embryonic stem cells
EZH2: Enhancer of zest 2
GATA4: Protein transcription factor 4 binding to GATA DNA sequence
HAT: Histone acetyl transferase
HDAC: Histone deacetylase
HDACi: Histone deacetylase inhibitor
H3K9Ac: Histone H3 acetylated at lysine 9
H3K9me2/3: Histone H3 bi- or trimethylated at lysine 9
H3K4me3: Histone H3 trimethylated at lysine 4
H3K27me3: Histone H3 trimethylated at lysine 27
5hmC: 5-hydroxymethylcytosine
HMGA: High-mobility group A
iPSCs: Induced pluripotent stem cells
IWP-1: Inhibitor of Wnt production 1
IWR-1: Inhibitor of Wnt response 1
LEF: Lymphoid enhancer binding factor
LncRNA: Long-noncoding RNA
LPR: Low-density lipoprotein receptor related
miR: microRNA
5mC: 5-Methylcytosine
MMP: Metalloprotease
MSCs: Mesenchymal stem cells
NKx2.5: NK2 homeobox protein 5
NuRD: Nucleosomal remodeling and deacetylase
OSKM: Oct4, Sox2, Klf4, cMyc
Osx: Osterix
P: Phosphate group
p21^cip1^: p21 CDK-interacting protein 1
p57^kip2^: p57 kinase inhibitor protein 2
PPAR: Peroxisome proliferator-activated receptor
PTEN: Phosphatase and tension homolog
PTMs: Posttranslational modifications
Rb: Retinoblastoma protein
RUNX: Runt-domain transcription factor
Sfrp2: Secreted frizzled related protein 2
SWI/SNF: Switch/sucrose nonfermentable
TCF: T-cell binding factor
Wnt: Wingless-related integration site.
